# Simulation Analysis of 3-D Airflow and Temperature Uniformity of Paddy in a Laboratory Drying Oven

**DOI:** 10.3390/foods13213466

**Published:** 2024-10-29

**Authors:** Changzhi Wang, Yongsheng Pei, Zhongqiu Mu, Lin Fan, Jian Kong, Guizhong Tian, Shiyuan Miao, Xiangyi Meng, Hai Qiu

**Affiliations:** 1 College of Mechanical Engineering, Jiangsu University of Science and Technology, Zhenjiang 212100, China; 221210201130@stu.just.edu.cn (C.W.); peiyongsheng@just.edu.cn (Y.P.); 231110201207@just.edu.cn (L.F.); kongjian1411969747@126.com (J.K.); tianshuanger@126.com (G.T.); mengxy@just.edu.cn (X.M.); 2 Nanjing Institute of Agricultural Mechanization, Ministry of Agriculture and Rural Affairs, Nanjing 210014, China; muzhongqiu@caas.cn; 3 School of Grain Science and Technology, Jiangsu University of Science and Technology, Zhenjiang 212100, China; 33729144@student.murdoch.edu.au; 4 School of Agricultural Science, College of Environmental and Life Sciences, Murdoch University, Perth, WA 6150, Australia

**Keywords:** paddy, multi-physics field simulation, airflow distribution, drying uniformity, convection drying

## Abstract

This study analyzed the effects of airflow characteristics on the temperature distribution and drying uniformity of paddy during convective drying. Simulations of the drying process with varying airflow inlet and outlet positions were conducted using COMSOL Multiphysics 6.1 software. The determination coefficient (*R*^2^) between the simulated data and experimental values of Sample1 (S1), Sample2 (S2), and Sample3 (S3) was calculated, and its average values were 0.964, 0.963, 0.963, and 0.967, respectively. This study demonstrates that the airflow direction and outlet location have a significant impact on the temperature uniformity of the paddy. The vortex structure generated by the obstruction of the sidewalls and paddy influences both the airflow and temperature distribution within the drying chamber. When the outlet was on the left side and the inlet airflow was in a vertical orientation (VO), the temperature distribution of the paddy exhibited higher temperatures in the edge regions and lower temperatures in the center, with a maximum temperature difference of around 16 °C. The time required for the temperature to reach equilibrium with the outlet positioned on the left was 28.6% shorter than with the outlets positioned in the center or on both sides. Moreover, the temperature uniformity of the three paddy samples was better under this condition. The developed model accurately reflected the paddy drying process. It could also be used to analyze the optimal heating uniformity, providing a technical basis for the design of grain dryers.

## 1. Introduction

Paddy is an essential staple food for 50% of the world’s population [1]. The global paddy cultivation area was recorded at approximately 160 million hectares [2], with worldwide production reaching around 760 million tons. Generally, the initial moisture content of post-harvest paddy exceeds 20% on a wet basis (w.b.), resulting in heightened susceptibility to damage from the growth of microorganisms, insects, and pests. Consequently, this vulnerability will shorten the product’s shelf life and escalate post-harvest losses [3]. Drying is the common and effective approach for the dehydration of paddy, and it helps maintain the post-harvest quality of paddy, minimize yield losses, and extend long-term storage [4]. Recently, convection drying (CD) has emerged as a traditional and conventional drying method for drying paddy and other crops in the food process industry. It offers several advantages such as simple operation, low cost, and direct heating and drying characteristics [5].

CD involves a complex nonlinear coupled problem due to the synergistic effects of multiple physical fields [6]. It encompasses the convective transfer of heat and momentum between hot air and materials, the diffusive transfer of heat and mass within materials, and phase changes in water [7]. Therefore, a comprehensive investigation into the formation and spatial distribution of the flow and high-temperature fields in CD is essential for elucidating their influence on the drying characteristics of paddy. Commonly, experimental analysis is an effective approach for investigating the drying process of materials. Nonetheless, most experiments entail a significant time investment, high costs associated with trial and error, and susceptibility to environmental influences [8]. Moreover, the visualization and detection of air temperature distribution and velocity fields during CD are constrained by the limitations of existing detection equipment. With the development of computer numerical simulation technology, numerical simulation can mitigate numerous repetitive experiments, shorten operation time, and simultaneously obtain data that are difficult to measure in experimental analysis. It is a low-cost and effective tool for exploring the drying characteristics of food products throughout the drying process [9].

At present, various mathematical models have been developed to investigate the heat and mass transfer of food products during CD. Simplification and appropriate assumptions for mathematical models are effective strategies to reduce modeling complexity and improve computational speed. By defining the parameters of the material, the temperature and moisture distribution of its simplified model can be analyzed. Onwude et al. [10] and Pei et al. [11] developed a two-dimensional axisymmetric geometry model to represent fruit and vegetable slices, considering both evaporation and convection occurring only at the material boundary. Zhao et al. [12], Wei et al. [13], and Chen et al. [14] established three-dimensional particle geometry models to represent brown paddy, corn, and peanuts, respectively. Obviously, the above studies ignored the characteristics and conditions of airflow in the drying chamber, without considering the impact of these conditions on the drying mechanisms of food products. In addition, considering the fluid characteristics, Selimefendigil et al. [15] studied the heat transfer and mass transfer processes of a moving porous moist object in a rectangular channel under laminar conditions. They found that the heat transfer coefficient was time-dependent and varied locally. However, variations in fluid and material properties make rectangular channel modeling inadequate for accurately representing the three-dimensional effects of inner sidewalls on airflow distribution conditions in real drying equipment. Additionally, the arrangement of the air inlets and outlets complicates the analysis of how variations in airflow distribution affect drying uniformity. Huo and Wang [16] studied the uniformity of velocity and temperature in the drying chamber by optimizing the number of wind cones and outlets. Wang et al. [17] analyzed the characteristics of airflow and temperature distribution inhomogeneity through simulations in the drying room. These above studies have demonstrated that the size and number of air inlets and outlets, along with wind speed, significantly impact drying uniformity in a drying room. However, there is limited comprehensive research on the airflow distribution characteristics in a drying chamber and their impact on the drying uniformity of food products.

During the CD process, the air inlets and outlets, along with the wall and material boundaries, generate a specific heat convection distribution within the drying chamber. These varying convection and temperature fields impact the heat and mass transfer characteristics of food products. Therefore, this study aims to develop a three-dimensional finite element CD model using the COMSOL simulation method. The formation and distribution characteristics of the airflow and temperature in the drying chamber were analyzed by adjusting the positions of the air inlets and outlets. The model also accounts for the blockage effect of the paddy boundary layer at various positions to evaluate the influence of different airflow field distributions on the drying uniformity of paddy.

## 2. Materials and Methods

### 2.1. Materials

In this study, paddy (Zhendao 668) was harvested from Jiangsu Province. The original samples were cleaned, and germinated and cracked paddy particles were removed to select uniform and whole paddy as the test samples. Then, the selected paddy was stored in sealed plastic bags and kept refrigerated in a refrigerator (SC/SD-332, Haier Refrigeration Division, Qingdao, China) at 4 °C for the experiment. The initial moisture content (*M*_0_) of paddy was 24.5 ± 0.2% on a wet basis (w.b.).

### 2.2. Drying Equipment and Experimental Design

**Figure 1:**
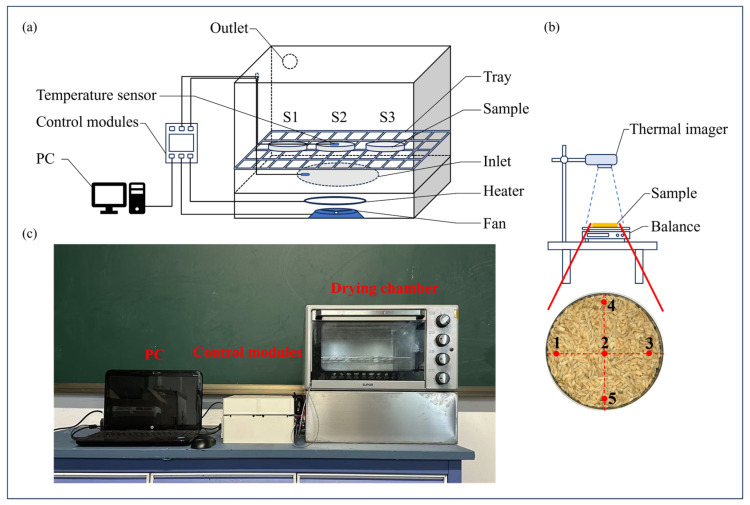
Physical and schematic diagram of CD system. (**a**) Schematic diagram of CD system. (**b**) Schematic of thermal imaging photo of the paddy. (**c**) Drying equipment physical diagram.

A custom-built laboratory CD system was used for the experiments, as illustrated in Figure 1. The dryer mainly consisted of an electric heater, thyristor, acquisition card, fan, and temperature sensor. The drying chamber is constructed of galvanized steel and has dimensions of 350 mm × 250 mm × 300 mm (L × H × W). Hot air was generated by an electric heater installed on one side of the drying chamber and was circulated into the chamber by a fan. In addition, the porous steel plates were installed at the inlet to ensure a more uniform airflow. The inlet airflow velocity was maintained at 1 m/s. The air temperature in the drying chamber and material temperature were continuously monitored using K-type temperature sensors (WRNT-035-2PBO, Shanghai Songdao Heating Sensor Co., Ltd., Shanghai, China). The output power of the electric heater was adjusted based on a comparison between the temperature in the drying chamber and the set temperature. Temperature data were captured using an NI data acquisition card (USB-6009, National Instrument Co., Ltd., Austin, TX, USA) equipped with analog input and analog output channels. An output signal was generated through the NI data acquisition card and transmitted to a thyristor to regulate the heating power, maintaining the temperature in the drying chamber at a set value. The pre-experiment indicated that the temperature control had an accuracy with an error of less than 0.1 °C.

Before the drying experiment, the paddy was removed from the refrigerator and acclimatized to ambient conditions at a room temperature of 20 ± 2 °C. The pre-experiment compared the drying time and basic physical qualities (the cracking rate) of paddy at four constant temperatures (50 °C, 60 °C, 70 °C, and 80 °C). The drying time of the paddy at 50 °C was the longest. Additionally, the cracking rate was lowest at 60 °C. The results show that a CD temperature of 60 °C produced the best quality. The paddy was evenly spread in a glass Petri dish with a diameter of 100 mm, forming a layer that was 10 mm thick. The arrangement was made as shown in Figure 1, with three glass Petri dishes spaced 10 mm apart. Rounded glass Petri dishes reduced the retention of airflow at the edges. In addition, the use of three separate round Petri dishes allowed for easy comparative study of the drying uniformity of different areas in an experiment. To improve airflow permeability, a stainless-steel wire mesh (300 mm × 350 mm × 0.3 mm) was used as a tray for glass Petri dishes. It was located 100 mm from the bottom of the drying chamber. Every 10 min of drying, the paddy sample was taken out of the drying system immediately to measure its surface temperature by a thermal imager (HM-TPK20-3AQF/W, HIKVISION, Hangzhou, China), and the weight of the paddy samples was measured using a digital balance (YP1002A, Shanghai Puchun Measuring Instrument Co., Ltd., Shanghai, China) to determine the MC value of the paddy during the drying process. Drying was stopped until the moisture content of the paddy reached 14.0 ± 0.2% (w.b.).

### 2.3. Evaluation of Drying Uniformity and Model Validation

The uniformity of the temperature distribution of the paddy layer significantly impacts the quality of the final products [5]. The collected infrared thermograms were processed using image analysis software. Figure 1 shows five measurement points marked in red on the surface of the paddy layer, which were selected to evaluate the temperature distribution. According to previous studies by Chen et al. [18] and Su et al. [19], the coefficient of uniformity (*COU*) is a suitable statistical parameter for quantifying drying uniformity.

(1) $$COU=\frac{\sqrt{\frac{1}{n}\sum_{i=1}^{n}{\left(y_{i}-\bar{y}\right)}^{2}}}{\bar{y}}$$

where *n* is the number of data, *y_i_* is the *i*th data point, and $\bar{y}$ is the average value of the dataset. *COU* is defined as the ratio of the standard deviation to the mean of the dataset, with lower *COU* values indicating better distributional uniformity [20].

The difference between the maximum and minimum temperatures (∆*T*) is used to describe the degree of thermal runaway due to uneven heating of the material [21,22].

(2) $$\Delta T=T_{\max}-T_{\min}$$

where *T_max_* and *T_min_* are the maximum and minimum temperatures within the specimens, respectively.

## 3. Mathematical Model Development

### 3.1. Problem Description and Assumptions

In a typical CD process, hot airflow continuously passes over the material’s surface, raising its temperature and removing surface moisture. Temperature and moisture gradients between the surface and interior generate a driving force for moisture migration, thereby effectively removing moisture from the entire material [23,24]. As shown in Figure 1, a stacked paddy layer is considered an isotropic multiphase porous medium because of its pore features. Moreover, the initial temperature and moisture of the paddy were uniformly distributed, and volume shrinkage due to water evaporation was considered negligible during the drying process. To examine the air distribution and flow characteristics in three-dimensional space during the drying process, a combination of computational fluid dynamics (CFD) and heat and mass transfer model were applied in this study. Therefore, a multi-physics field model was developed to describe the drying process of paddy. This model encompasses fluid flow, heat conduction, and mass transfer to investigate the effects of air distribution and flow within the drying chamber on the drying characteristics of paddy.

### 3.2. Physical Model

**Figure 2:**
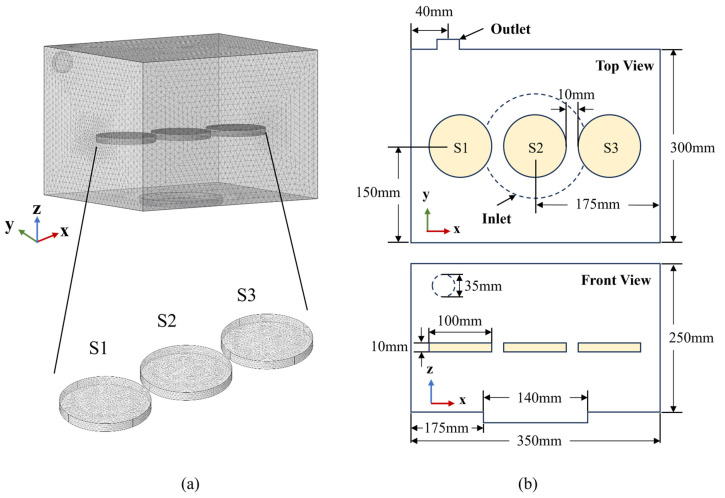
Three-dimensional computational domain for the CD simulation and their boundaries. (**a**) The air flow direction is vertical, and the outlet location is on the left side. (**b**) Computation of the top view and the front view of the domain.

In this study, a three-dimensional multi-physics field model was established to evaluate the effects of airflow conditions on paddy drying, including fluid flow and heat and mass transfer within the material. The CD equipment was simplified into a 1:1 scale three-dimensional geometrical model, consisting of the air–fluid domain of the drying chamber and the computational domain of the paddy layer, as depicted in Figure 2a. The paddy layer was considered a porous medium and marked as Sample1 (S1), Sample2 (S2), and Sample3 (S3) at the three locations illustrated in Figure 2b.

### 3.3. Governing Equations

#### 3.3.1. Heat and Mass Transfer Equations

According to Fourier’s law [25], heat transfer in a thin layer of paddy is described by the energy balance equation presented in Equation (3) for CD.

(3) $$\rho C_{p}\frac{\partial T}{\partial t}+\rho C_{p}u\nabla T=\nabla \cdot \left(k\nabla T\right)$$

where *T* is the temperature (°C) of the sample at time *t*, *ρ* is the density of the sample (kg/m^3^), *C_p_* is the specific heat of the material (J·kg^−1^K^−1^), and *k* is the thermal conductivity of the material (W·m^−1^K^−1^).

Moisture transfer within the thin layer of paddy during CD follows Fick’s diffusion law, as described by Equation (4):

(4) $$\frac{\partial c}{\partial t}+\nabla \cdot (-D_{\mathrm{eff}}\nabla c)=0$$

where *c* is the instantaneous moisture concentration (mol/m^3^), *t* is time (s), and *D_eff_* is the effective moisture diffusivity (m^2^/s).

The moisture concentration *c* is related to the wet basis moisture content (*M_wb_*) according to Equation (5) [26,27].

(5) $$c=\frac{M_{wb}\rho_{m}}{M_{H_{2}O}}$$

where *M_wb_* is the moisture content of the dried paddy (kg/kg, w.b.), *ρ_m_* is the material density of paddy (kg/m^3^), and $M_{H_{2}O}$ is the molecular weight of vapor (kg/mol).

#### 3.3.2. Initial and Boundary Conditions

The finite element modeling of paddy was performed using the input parameters specified in Table 1. Convective heat exchange between the paddy and the hot air occurred at the surface during CD [10]. The initial and boundary conditions of heat transfer are expressed as Equations (6) and (7).

(6) $$T=T_{0},\;\mathrm{at}\;t=0$$

(7) $$n\cdot \left(k\nabla T\right)=h_{t}\left(T_{air}-T\right)-h_{m}\rho \left(M_{db}-M_{e}\right)H_{vap}$$

where *T*_0_ is the initial temperature of the material (293.15 K), *t* is the time (s), *h_m_* is the mass transfer coefficient (m/s), *h_t_* is the heat transfer coefficient (W·m^−2^K^−1^), Me is the equilibrium moisture content (kg/kg, d.b.), and *T_air_* is the drying air temperature (°C). The latent heat of evaporation of water (*H_vap_*) is given by Equation (8) [28]:

(8) $$H_{vap}=2503000-2386\left(T_{air}-273.15\right);273.15<T\left(K\right)<533.15$$

The initial condition for the mass transfer equation is provided by Equation (9). The initial inlet velocity is given as Equation (10). The bottom of the paddy in contact with the tray is assumed to be a no-flux boundary condition, as specified by Equation (11). In contrast, external forced convection is applied to the other surfaces of the paddy, as expressed by Equation (12):

(9) $$c=c_{0},\;\mathrm{at}\;t=0$$

(10) $$\vec{u}=\vec{u_{0}},\;\mathrm{at}\;t=0$$

(11) n·−D∇c=0

(12) $$n\cdot -D\nabla c=h_{m}\left(c_{e}-c\right)$$

where *c* is the moisture concentration (mol/m^3^), $\bar{u}$ is the air velocity vector (m/s), and *c_e_* is related to the moisture content on a wet basis. *D* is the moisture diffusivity of paddy (m^2^/s).

The airflow temperature and airflow speed were set at the inlet, while a zero-static pressure was applied at the outlet of the computational domain. A Nono-slip boundary condition ($\bar{u}$ = 0) was also used for the dryer’s walls and for the paddy [29,30].

#### 3.3.3. Heat and Mass Transfer Coefficient Calculation

The heat and mass transfer coefficients at the sample boundaries mainly depend on the sample geometry and the fluid flow type. In this study, the thin layer of paddy was designed as a cylindrical porous structure, and the convective heat transfer coefficient under forced convection was calculated using the empirical formula provided in Equation (13).

(13) $$h_{t}=\frac{Nuk_{air}}{R_{eq}}$$

where *Nu* is the Nusselt number, *k_air_* is the thermal conductivity of air (W·m^−1^K^−1^), and *R_eq_* is the half-thickness of the thin layer of paddy (m). The Nusselt number (*Nu*) was obtained using the equation below, which is related to the Reynolds (*Re*) and Prandtl (*Pr*) numbers. *Re* and *Pr* can be expressed by Equations (15) and (16), respectively [31,32].

(14) $$Nu=2+0.552{\left(Re\right)}^{\frac{1}{2}}{\left(Pr\right)}^{\frac{1}{3}}$$

(15) $$Re=\frac{\rho_{air}v_{air}R_{eq}}{\mu_{air}}$$

(16) $$Pr=\frac{C_{pair}\mu_{air}}{k_{air}}$$

where *ρ_air_* is the air density (kg/m^3^), *v_air_* is the drying air velocity (m/s), *μ_air_* is the dynamic viscosity of air (Pa·s), and *C_pair_* is the specific heat of air (J·kg^−1^K^−1^).

The mass transfer coefficient is calculated from the Sherwood number (*Sh*) as shown in Equation (17) [33].

(17) $$Sh=\frac{h_{m}R_{eq}}{D_{air}}=2+0.552{\left(Re\right)}^{\frac{1}{2}}{\left(Sc\right)}^{\frac{1}{3}}$$

where *D_air_* is the diffusivity of water in air (m^2^/s) and *Sc* represents the dimensionless Schmidt number, calculated by Equation (18).

(18) $$Sc=\frac{\mu_{air}}{\rho_{air}D_{air}}$$

**Table 1:** Various parameters used in momentum, heat, and mass transfer modeling.

Parameters	Value or Expression	Unit	Reference
The initial temperature of paddy, *T*_0_	20	°C	This work
Drying air temperature, *T_air_*	60	°C	This work
The initial temperature of air, *T_a_*_0_	25	°C	This work
Molecular weight of vapor, $M_{H_{2}O}$	0.01802	kg/mol	[34]
Activation energy, *E_a_*	29.85017	kJ/mol	[27]
The initial moisture content of paddy, *M_wb_*	0.245	kg/kg, (w.b.)	This work
Density of paddy, *ρ_m_*	1716	kg/m^3^	This work
Diffusion coefficient of paddy, *D*	$7.98\times 10^{-6}\times \exp\left(-\frac{E_{a}}{RT}\right)$	m^2^/s	[27]
Specific heat capacity of paddy, *Cp_m_*	$1.180+3.766M_{\mathrm{wb}}$	kJ·kg^−1^K^−1^	[35]
Thermal conductivity of paddy, *k_m_*	$\left(0.0637+0.0958M_{\mathrm{db}}\right)/\left(0.656-0.475M_{\mathrm{wb}}\right)$	W·m^−1^K^−1^	[35]
Density of air, *ρ_air_*	$8.666\times 10^{-6}T^{2}-4.318\times 10^{-3}T+1.288$	kg/m^3^	[27]
Thermal conductivity of air, *k_air_*	$-2.401\times 10^{-8}T^{2}+7.554\times 10^{-5}T+0.02364$	W·m^−1^K^−1^	[27]
Specific heat capacity of air, *C_pair_*	$4.835\times 10^{-4}T^{2}-0.02218T+1007$	kJ·kg^−1^K^−1^	[27]
Dynamic viscosity of air, *μ_air_*	$3.238\times 10^{-11}T^{2}+4.839\times 10^{-8}T+1.73\times 10^{-5}$	Pa·s	[27]
Diffusivity of water in air, *D_air_*	$3.229\times 10^{-10}T^{2}+1.557\times 10^{-7}T+2.089\times 10^{-5}$	m^2^/s	[27]

### 3.4. Airflow Modeling

Turbulence is prevalent in most studies of processes, especially within the boundary layer at the surface of the material. The Reynolds-Averaged Navier–Stokes (RANS) equations are employed to simulate forced convection airflow in the drying chamber, owing to their effectiveness in modeling wall-bounded boundary layer flows [36]. To apply the turbulence model, the following assumptions were made: (i) thermal equilibrium of the hot air is maintained throughout the process; (ii) the single-phase mixture in the air is diluted, allowing for the neglect of Soret and Dufour effects; (iii) only forced convection is considered, with buoyancy effects excluded. The k-ε turbulence model is widely used for describing turbulent transport, including heat transfer problems. It can handle a large range of wall boundaries and free shear flows [37]. The standard k-ε turbulence model was selected in this study and was obtained by Equations (19) and (20):

(19) $$\rho \frac{\partial u}{\partial t}\rho \left(u\cdot \nabla \right)u=\nabla \cdot \left[-pI+Κ\right]+F$$

(20) $$Κ=\left(\mu +\mu_{t}\right)\left(\nabla u+{\left(\nabla u\right)}^{t}\right)-\frac{2}{3}\left(\mu +\mu_{t}\right)\left(\nabla u\right)I-\frac{2}{3}\rho \kappa I$$

where *ρ* is the density of the fluid (kg/m^3^); ***u*** is the fluid velocity (m/s^2^); *p* is pressure (Pa); ***F*** is the volume force, the external force acting on the fluid; K is the viscous force; and *μ* is the hydrodynamic viscosity.

The equations of turbulent kinetic energy and turbulent dissipation rate are also included [38]. The turbulent kinetic energy equation is calculated using Equation (21).

(21) $$\frac{\partial }{\partial t}\left(\rho \kappa \right)+\nabla \cdot \left(\rho \vec{v}\kappa \right)=\nabla \cdot \left(\frac{\mu_{t}}{\sigma_{\kappa }}\nabla \kappa \right)+G_{\kappa }-\rho \varepsilon$$

The dissipation rate equation is determined by Equation (22).

(22) $$\frac{\partial }{\partial t}\left(\rho \varepsilon \right)+\nabla \cdot \left(\rho \vec{v}\varepsilon \right)=\nabla \cdot \left(u+\frac{\mu_{t}}{\sigma_{\varepsilon }}\nabla \varepsilon \right)+\frac{\varepsilon }{\kappa }(C_{1}G_{\kappa }-C_{2}\rho \varepsilon )$$

By combining Equations (21) and (22), the turbulent viscosity (*μ_t_*) can be expressed as follows:

(23) $$\mu_{t}=\rho \frac{C_{\mu }\kappa^{2}}{\varepsilon }$$

where *κ* is the turbulent kinetic energy (m^2^/s^2^); *ε* is the dissipation rate (m^2^/s^3^); *ρ* is the density (kg/m^3^), $\vec{v}$ is the velocity (m/s); *G_κ_* is the production of turbulent kinetic energy (kg·m^−1^s^−3^); C*_μ_*, C_1_ and C_2_ are the empirical constants in the turbulence model; and *σ_κ_* and *σ_ε_* are the turbulent Prandtl numbers for the kinetic energy and the dissipation rate, respectively. Standard values of the model constants of the k-ε model used in the model are referred from Woo Park et al. [39]:

$$C_{\mu }=0.09,C_{1}=1.44,C_{2}=1.92,\sigma_{k}=1.0,\sigma_{\varepsilon }=1.3$$

### 3.5. Factors Affecting Drying Uniformity

**Figure 3:**
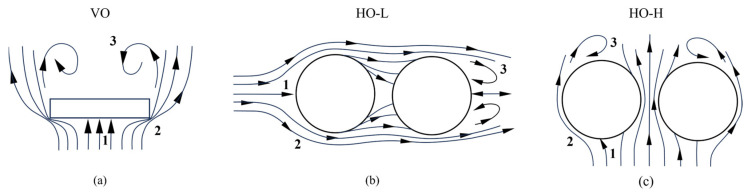
Schematic diagram of various airflow directions and sample placements. Arrows show the direction of the airflow stream. 1, 2, and 3 indicate stagnant area, separation area, and wake area, respectively. (**a**) Vertical airflow. (**b**) Horizontal airflow with the sample aligned parallel to the airflow. (**c**) Horizontal airflow with the sample aligned perpendicular to the airflow.

During the CD process, the airflow is obstructed to form turbulence. Variations in turbulence location and turbulence values reveal an uneven flow field distribution. Larger turbulence differences correspond to great inhomogeneity in the internal flow field. It also causes inhomogeneity in paddy drying. Therefore, three different airflow directions were investigated in this study, as shown in Figure 3. Figure 3a shows that the airflow direction is in a vertical orientation (VO). Figure 3b,c illustrate the airflow in the horizontal orientation (HO). The material’s placement can influence both airflow diversion and the uniformity of drying across different locations of the paddy layers. Two types of airflow under the horizontal orientation have been distinguished. The first type features the air inlet on the right side of the drying chamber, with the paddy arranged longitudinally along the airflow direction (HO-L). The second type has the air inlet at the back of the drying chamber, with the paddy arranged horizontally (HO-H). Referring to Figure 3 and examining the airflow trajectories, vertical and horizontal airflow conditions have the same features [40]: When the airflow is obstructed, the central part of the front side becomes a stagnation area (locus 1: stagnant area). The airflow at the front edge of the obstacle flows outward, forming a separation area (locus 2: separation area). Moreover, the turbulence effects extend past the trailing edge and deep into the wake (locus 3: wake area). These factors will affect localized heat and mass transfer within the paddy layer.

The airflow distribution in the drying chamber is affected by both the hot air outlet and the air outlet. The position of the air outlet may cause deformation of the turbulent vortex structure [38]. Therefore, four outlet positions (left, right, center, and left and right sides) at the back of the drying chamber were chosen to investigate the impact of the air outlet’s location on the airflow field and the resulting drying inhomogeneity of the paddy layer.

### 3.6. Simulation Procedure

**Figure 4:**
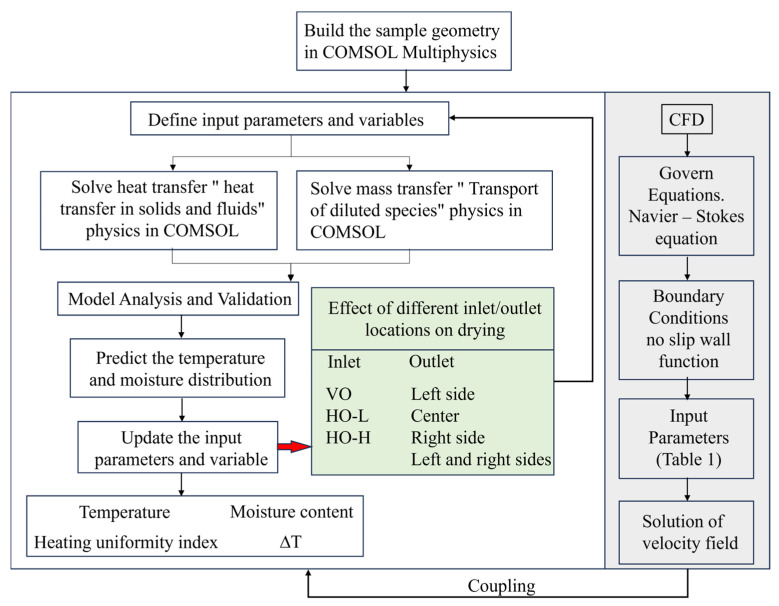
The flow chart of the overall model simulation strategy.

The simulation was conducted using COMSOL Multiphysics software (version 6.1, COMSOL Inc. Wuhan, China), which effectively handles the complexity of flow field data and coupled heat and mass transfer modeling for analyzing temperature and moisture distribution. The flow chart of the simulation strategy for solving the heat and mass transfer equations using CFD coupling is given in Figure 4. The transient calculation of CFD demonstrates a dependency on time, while steady-state simulations can reduce the computational load. To balance accuracy and computational efficiency, the CFD module was calculated using “steady-state RANS k-ε turbulence”. In addition, to study the temporal variations in the temperature and moisture content of the materials within the chamber, “solid and fluid heat transfer (ht)” and “dilute substance transport (tds)” used transient studies.

Mesh sensitivity tests were performed with different types of meshes (normal, fine, ultra-fine, and very fine) to confirm that the simulation results are not dependent on the mesh types. The accuracy of the model calculations was considered independent of grid size when the difference between the maximum, minimum, and average temperatures between adjacent grid sets was less than 0.1% [41]. Considering the model accuracy and computational efficiency, a normal mesh was chosen for the fluid computational domain. The maximum and minimum element sizes are 1.9 and 0.568, respectively. The solid computational domain employs a very fine mesh, with the maximum and minimum element sizes being 0.7 and 0.007, respectively. The total number of cells is 167,522. The simulations were carried out on a local computer Intel(R) Xeon(R) Gold 6130 CPU @ 2.10GHz (NVIDIA Quadro P2200) with an installed RAM of 128GB, running under Windows 10 (64 bit).

### 3.7. Model Validation and Analysis

The measured temperature and moisture content of the paddy layer were compared to the simulated values based on the coefficient of determination (*R*^2^) and root mean square error (*RMSE*) [42,43]. Higher *R*^2^ values and lower *RMSE* values indicate a better fit for the simulation model. *R*^2^ and *RMSE* were obtained using Equations (24) and (25):

(24) $$R^{2}=1-\left[\frac{\sum_{i=1}^{n}{\left(X_{sim,i}-X_{exp,i}\right)}^{2}}{\sum_{i=1}^{n}{\left(X_{sim,i}-\bar{X_{exp}}\right)}^{2}}\right]$$

(25) $$RMSE=\sqrt{\frac{1}{N}\sum_{i=1}^{n}{\left(X_{sim}-X_{exp}\right)}^{2}}$$

where *X_sim_* and *X_exp_* are the simulated and experimental values, *n* is the total number of measurement points, and *i* is the *i*th data point.

## 4. Discussion

### 4.1. Model Validation

**Figure 5:**
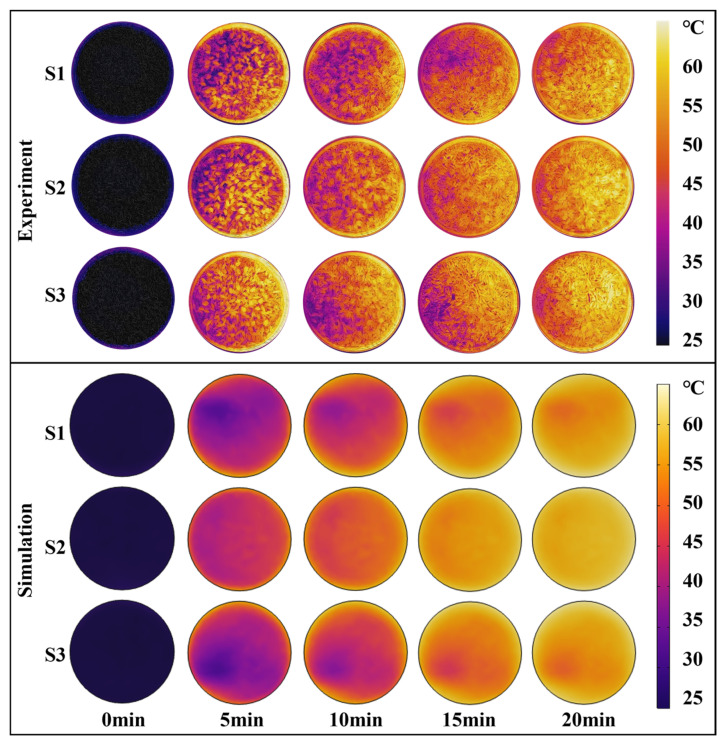
Comparison of the simulated and experimental surface temperature of the paddy layer during CD at 60 °C.

Figure 5 presents a comparison of the simulated and experimental surface temperature distributions of the paddy layer during the first 20 min of the drying process at 60 °C. The average temperature of the paddy increased throughout the drying period, rising from an initial value of 20.1 ± 0.1 °C to 54.6 ± 1 °C within 20 min, according to the experimental results. The simulation results closely matched the temperature distribution trend observed in the actual infrared thermal images. The surface temperature distribution of the paddy at the three locations (S1, S2, and S3) was uneven. The edge temperatures were higher than those of the central surface. Notably, a significant temperature difference was observed across the paddy layer surface at 5 min of drying. The temperature differences for the three samples were 16.8 °C, 12.6 °C, and 13.3 °C, respectively. The original flow field changes when the airflow boundary is disturbed by relatively obstructed protrusions. In addition, the different locations of the air outlets lead to uneven airflow distribution and uneven convective heat transfer on the sample surface, resulting in significant temperature differences.

As the drying time increases, the temperature of the paddy gradually rises, and the temperature distribution on the surface becomes more uniform. Specifically, when the drying time reached 20 min, the maximum temperatures measured for S1, S2, and S3 were 56.5 °C, 58.3 °C, and 58.4 °C, respectively, while the minimum temperatures were 47.9 °C, 49.3 °C, and 48.1 °C, respectively. Wei et al. [44] investigated that during radio frequency-assisted hot air drying, the highest temperatures of corn kernels were found at the corners and edges of the container and the lowest temperatures were found in the center. In the present study, paddy dried faster at the edges of the vessel than at the edges and center of the vessel. This uneven drying was a consequence of airflow and temperature inhomogeneity.

**Figure 6:**
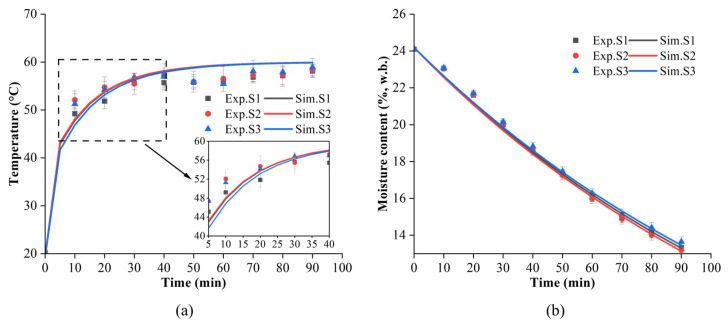
Comparison of simulated and experimental temperature and moisture content of the paddy layer during CD at 60 °C. (**a**) Temperature versus time curves. (**b**) Moisture content versus time curve.

Figure 6 illustrates the changes in the simulated and experimental temperature and MC profiles of S1, S2, and S3 during CD at 60 °C. As can be seen in Figure 6a, the temperature of the paddy increased sharply in the early stage of drying. After 50 min, the temperature of the paddy and air reached a thermal equilibrium state and remained stable. Statistical approaches were utilized to evaluate the simulation model against the experimental data (Table 2). The statistical results indicated that the *RMSE* and *R*^2^ values for the temperature of the paddy samples were 2.155 and 0.967, respectively. The deviation between the simulated and experimental temperatures was greater compared to the MC. The main factors contributing to temperature deviation are as follows: (1) significant temperature differences between the paddy and the surrounding environment during the sampling and infrared imaging period, resulting in heat loss [45]; (2) greater heat exchange and increased heat loss at higher material temperatures during the later stages of drying in the actual experiment; (3) discrepancies between the model’s physical parameters and actual values during the drying process [44,46]. The *R*^2^ for MC between the simulation and the experimental results exceeded 0.99, indicating that the proposed model accurately describes the process of paddy CD.

### 4.2. Flow Field Distribution for Different Airflow Directions

**Figure 7:**
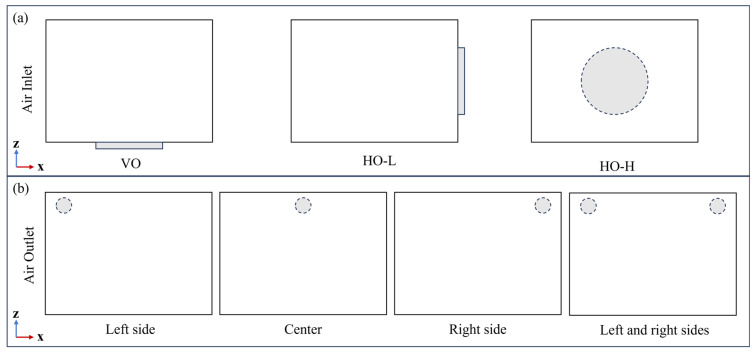
Schematic diagram of the air inlets (**a**) and outlets (**b**) from the main view under different airflow directions.

The airflow pattern that contacts the paddy varies with the position of the air inlet. In this simulation study, as shown in the schematic diagram of the main view in Figure 7, three airflow patterns of VO, HO-L, and HO-H were established by positioning the air inlet at the bottom, left side, and back of the drying chamber, respectively. Simultaneously, four air outlet configurations at the back of the drying chamber were analyzed, as shown in Figure 7b. Their positions were at the left side, center, right side, and both left and right sides, and they were used to assess the airflow distribution characteristics within the drying chamber. To understand the three-dimensional flow characteristics of the fluid and the blockage effect caused by the paddy layer, four observation views were selected based on the three-dimensional coordinate section direction: X = 175 mm, Y = 150 mm, Z = 105 mm, and cut-away view at the outlet position.

#### 4.2.1. Flow Field Distribution Analysis of VO

**Figure 8:**
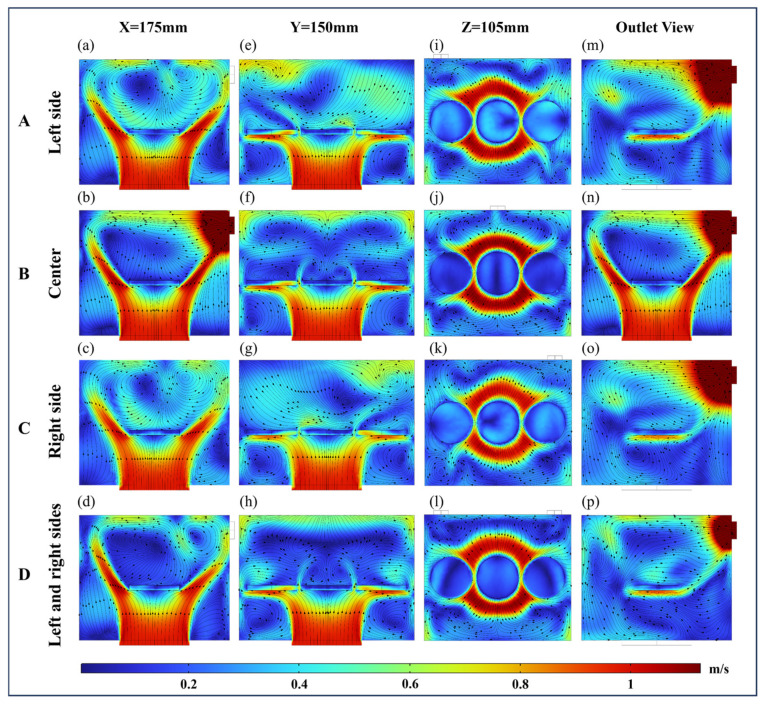
Velocity distributions at different outlets with the airflow direction of VO. Arrows show the direction of the airflow stream. A, B, C, and D indicate that outlets are located on the left, center, right, and left and right sides, respectively. (**a**–**d**) Coordinate section directions for X = 175 mm. (**e**–**h**) Coordinate section directions for Y = 150 mm. (**i**–**l**) Coordinate section directions for Z = 105 mm. (**m**–**p**) Outlet views.

Figure 8 displays the velocity distribution of airflow within the drying chamber at different outlet positions under the condition of the inlet airflow direction of VO. The airflow inlet is located at the bottom center of the dryer and the outlet location is located at its back. The outlet positions A and C are symmetrical, resulting in a mirror-image distribution of airflow at both outlets. The initial airflow velocity entering the drying chamber was 1 m/s. Initially, when the airflow contacted the paddy layer, it caused flow stagnation or separation. The airflow stagnated at the bottom of S2, with a velocity ranging between 0.2 and 0.4 m/s. Then, a portion of the airflow flowed from the gaps between the three samples toward the surface above the samples. From the view at Z = 105 mm, the velocity at the leading edge of S2 was 0.6 m/s. Another portion flowed along S1 and S3 toward the sidewalls of the drying chamber. The airflow then diffused along the sidewalls, creating a radial diffusion flow pattern. From the outlet view, the airflow velocity at the outlet exceeded 1 m/s as the transition from obstructed to unobstructed airflow caused a rapid increase in the outlet airflow velocity [16].

The vortex structure that formed due to the wake effect resulted in low airflow velocities at the sample surface. Observed from the viewpoint at X = 175 mm, a counter-rotating vortex structure appeared above the paddy when air outlets were located at the left, right, and both sides (Figure 8a,c,d) [47]. In contrast, when the outlet was positioned in the center (Figure 8e), the airflow above the paddy surface converged toward the outlet without forming a pair of counter-rotating vortex structures. The average surface velocity of the samples at the center and both sides outlets was 0.2 m/s, while it reached 0.4 m/s at the left and right outlets. From the Z = 105 mm viewpoint, it was observed that when the outlet was located on the left side (Figure 8i), the airflow velocity on the paddy surface was the highest among the four outlet positions, averaging 0.4 m/s.

#### 4.2.2. Flow Field Distribution Analysis of HO-L

**Figure 9:**
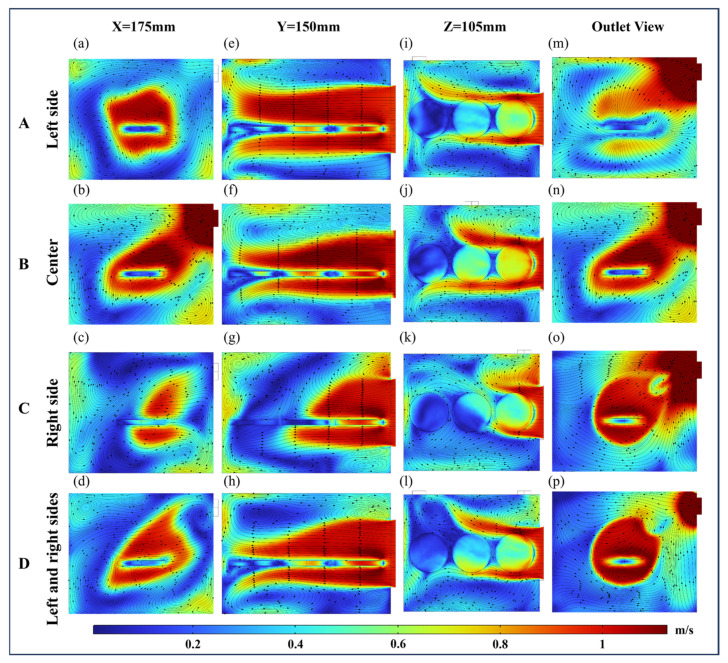
Velocity distributions at different outlets with the airflow direction of HO-L. Arrows show the direction of the airflow stream. A, B, C, and D indicate that outlets are located on the left, center, right, and left and right sides, respectively. (**a**–**d**) Coordinate section directions for X = 175 mm. (**e**–**h**) Coordinate section directions for Y = 150 mm. (**i**–**l**) Coordinate section directions for Z = 105 mm. (**m**–**p**) Outlet views.

Figure 9 illustrates the effects of the four outlet positions on the airflow distribution and velocity in the drying chamber with HO-L inlet airflow. The airflow was obstructed or bypassed by the edge, resulting in a reduction in velocity. This caused the airflow velocity above the paddy surface to decrease with increasing distance. In addition, this led to an uneven flow field on the surface of the paddy thus affecting the heat transfer efficiency. Therefore, among the four outlet positions, S3 was directly impacted by the airflow, resulting in high kinetic and thermal energy transfer efficiency. The airflow over S2 shifted toward the outlet, while stagnation zones appeared near S1. Additionally, the airflow velocity between S1 and S2 consistently remained below 0.4 m/s.

The cloud plots of each cross-section reveal that variations in the outlet positions significantly influenced the airflow distribution. The airflow converged toward the outlet position, resulting in an increase in velocity gradient as the outlet position moved from left to right. When the outlet was positioned on the right side, observations at Y = 150 mm and Z = 105 mm showed that the outlet location significantly impacted both S1 and S2. Furthermore, when the airflow reached the surface of S2, it underwent significant deflection. When the outlet was positioned on the right side, airflow recirculation above S1 formed four vortices, causing significant energy loss in the hot air and reducing airflow velocity around S1 to below 0.4 m/s. Meanwhile, the velocity gradient on the surface of S2 increased, ranging from 0 to 0.6 m/s.

#### 4.2.3. Flow Field Distribution Analysis of HO-H

**Figure 10:**
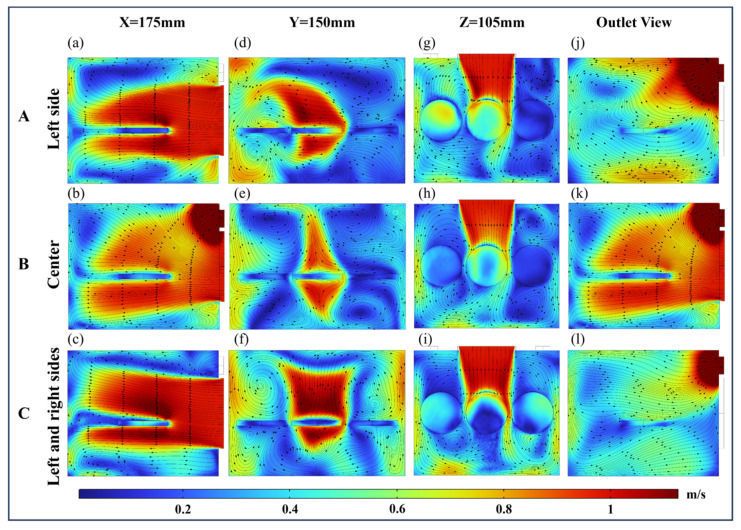
Velocity distributions at different outlets with the airflow direction of HO-H. Arrows show the direction of the airflow stream. A, B, and C indicate that outlets are located on the left, center, and left and right sides, respectively. (**a**–**c**) Coordinate section directions for X = 175 mm. (**d**–**f**) Coordinate section directions for Y = 150 mm. (**g**–**i**) Coordinate section directions for Z = 105 mm. (**j**–**l**) Outlet views.

Figure 10 shows the airflow velocity distribution at various outlets with the HO-H airflow direction. Since the inlet and outlet were located at the back side, the paddy samples were arranged perpendicular to the horizontal airflow, as shown in Figure 3c. The width of the drying chamber was 300 mm, resulting in a relatively short airflow path. After the airflow struck the chamber wall, it rapidly diffused along the chamber surface, leading to higher airflow velocity near the walls compared to the interior [38]. Since the left and right outlet positions were mirror images, their flow field distributions also mirrored each other. The cross-sectional views at Y = 150 mm and Z = 105 mm provided a clearer observation of how different outlet positions affected the flow field distribution and the airflow around the paddy. When the outlet was positioned on the left, the airflow converged toward it, and the shorter airflow path resulted in a smaller velocity gradient. Additionally, the poor airflow around S3 caused the airflow velocity around S1 to be 0.4 m/s higher than that around S3 (Figure 10d), resulting in a faster heating rate for S1 compared to S3. In contrast, when the outlet was positioned on the left and in the center, the average velocities of the surfaces of S1 and S3 were higher when the outlets were located on both sides.

Moreover, the velocity of the gaps in the three samples was influenced by the outlet position, leading to a deflection of the wake flow. When the outlets were in the center and on both sides, the deflected flow created stagnant regions in front of and behind S1 and S3. This led to reduced velocity and uneven distribution, negatively impacting convective heat transfer.

### 4.3. Temperature Distribution Under Different Airflow Directions

#### 4.3.1. Temperature Distribution Analysis of VO

**Figure 11:**
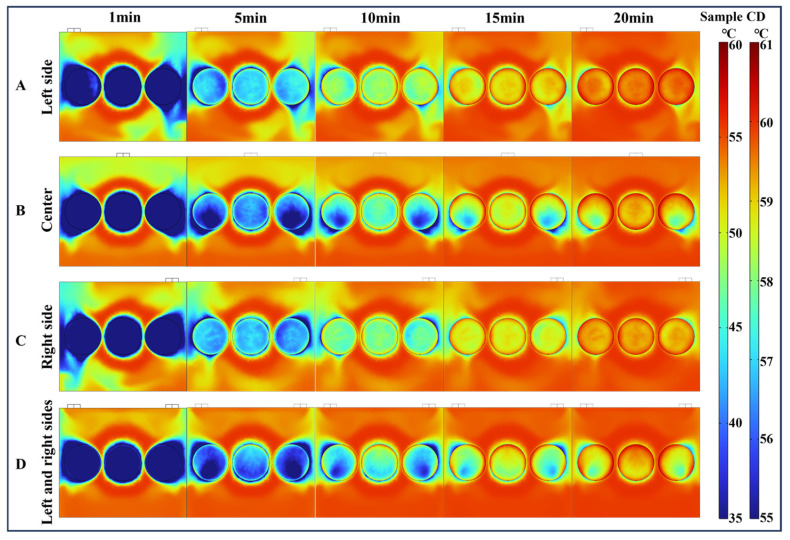
Temperature distribution in the drying chamber under different outlet locations with VO inlet airflow at 60 °C during the CD process. A, B, C, and D indicate that outlets are located on the left, center, right, and left and right sides, respectively.

Figure 11 shows the effect of different outlet locations on the temperature field distribution at 60 °C VO airflow. In the drying process at the four outlet positions, the temperature distribution within the drying chamber was not uniform. Throughout the 20 min CD process, the temperature of the paddy gradually increased with time. The hot air in contact with the surface of the paddy effectively transfers heat, leading to moisture evaporation and a rapid increase in the surface temperature of the paddy [48]. After 1 min of drying, the temperature at the edge of the paddy was higher than the surface center temperature. This is because the thermal conductivity of the tray is higher than that of the paddy. The tray heated up quickly and conducted heat to the paddy, creating a synergistic effect with the convective heat transfer from the air, which resulted in a rapid increase in temperature at the edges. The velocity of the separated regions at the edge of the paddy was higher than the velocity at the center of the paddy surface, which also accelerated the temperature rise at the edge of the paddy [49].

A comparison of the drying process with the four outlet locations revealed that the surface temperatures were more uniform under the left-and-right-outlet condition. After 10 min of drying, the temperature distribution on the surfaces of samples with outlets on the left and right was more uniform than that of samples with outlets at the center and on either side. It was observed that at 10 min of drying, the surfaces of S1 and S3 with outlets in the middle and on either side formed a dead zone with lower airflow velocities and, consequently, lower surface temperatures. The surface temperature differences between S1 and S3 under the center outlet condition were 20.27 °C and 20.31 °C. With outlets on both sides, the surface temperature differences between S1 and S3 were 20.49 °C and 19.78 °C, respectively. These differences resulted from varying outlet positions. Moisture on the windward side of the paddy surface evaporated, and the airflow carried the water vapor. When the airflow reached the tail region and dead zone, the reduction in velocity and temperature led to an increased temperature difference [49].

#### 4.3.2. Temperature Distribution Analysis of HO-L

**Figure 12:**
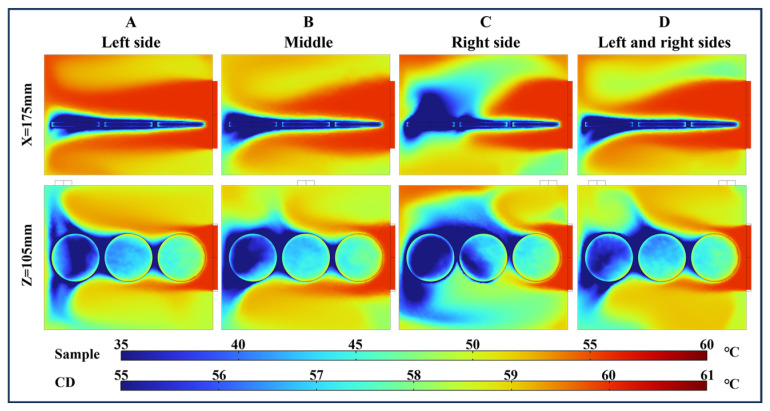
Temperature distribution with different air outlet positions under HO-L at 60 °C after 5 min of drying. A, B, C, and D indicate that outlets are located on the left, center, right, and left and right sides, respectively.

Figure 12 shows the temperature distribution with different air outlet locations after 5 min of drying, with the airflow direction indicated as HO-L. At 5 min of drying, the temperature difference between the samples in the graph was more pronounced. The airflow first impacted the surface of S3, which had a larger contact area with the hot air. Following the direction of the airflow, the temperatures of S3, S2, and S1 gradually decreased. The air underwent convective heat transfer, along with the evaporation of moisture from the paddy. The heat energy of the air was gradually consumed, resulting in a gradual reduction in temperature [50]. Moreover, the surfaces of the paddy were all areas of airflow separation, resulting in low airflow velocities on the surfaces of the paddy.

The temperature distributions with four different outlet locations of X = 175 mm revealed a significant low-temperature area above the surface of S1 when the outlet was located on the right. Combined with the velocity distribution (Figure 9g), there was a loss of kinetic energy in the airflow on the surface of S1. The low airflow velocity on the surface of S1 was a key factor contributing to the large area of low temperature. Looking at the temperature distribution from Z = 105 mm, the low-temperature area in the cavity was larger when the outlet was located on the right. Moreover, the surface temperature distribution of S2 was compared with the remaining three models, and the temperature difference was more obvious, with the temperature in its wake region being lower than 55 °C.

#### 4.3.3. Temperature Distribution Analysis of Airflow of HO-H

**Figure 13:**
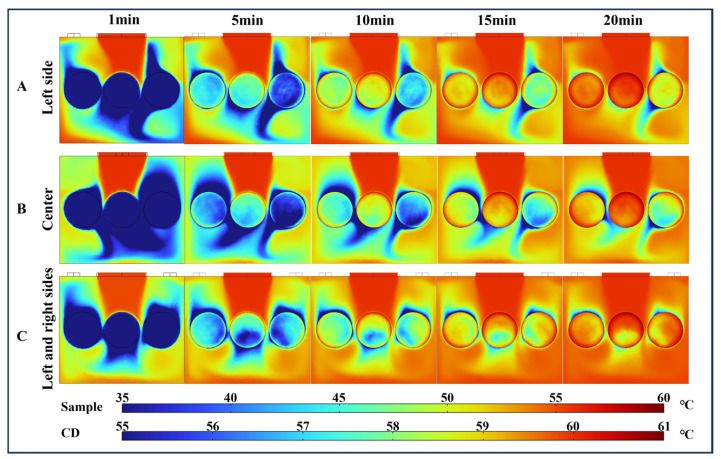
Temperature distribution with different outlet positions under HO-H, with a temperature of 60 °C. A, B, and C indicate that outlets are located on the left, center, and left and right sides, respectively.

Figure 13 shows the temperature distribution of the inlet and outlet positions of the airflow located at the back of the drying chamber. During the first 20 min of drying, the difference in outlet position had a significant effect on the temperature distribution around the three samples. The main effects included the temperatures at the gaps and edges of the paddy for the three samples, the windward surface temperatures of S1 and S3, and the tail temperature of S2. The average surface temperatures of S2 were 56.87 °C, 55.28 °C, and 53.33 °C at 20 min, respectively. However, the temperatures of S1 and S3 were affected by the air outlet, resulting in a temperature difference.

When the outlet was located on the left, the low-temperature wake of S2 and S3 shifted noticeably to the left. The low-temperature area on the windward side of S1 at the left outlet was smaller than that at when the outlet was at the center and on both sides. As the airflow from the gap between S1 and the drying sidewall flowed toward the intermediate outlet position, it resulted in a low temperature on the windward side of S1 with the outlet at the center. When the outlets were located on both sides, the tail temperatures of S1 and S3 were more symmetrical. Stagnant airflow near the wall region caused a buildup of heat in the airflow, reducing energy dissipation and resulting in higher temperatures at the trailing edges of S1 and S3 [51]. In comparison to the flow field distribution shown in Figure 10c, the temperature inhomogeneity on the surface of S2 was due to the shorter inlet airflow length. After hitting the wall, the airflow flowed directly along the upper wall to the exit position.

### 4.4. Effect of Different Parameters on Drying Uniformity of Paddy

**Figure 14:**
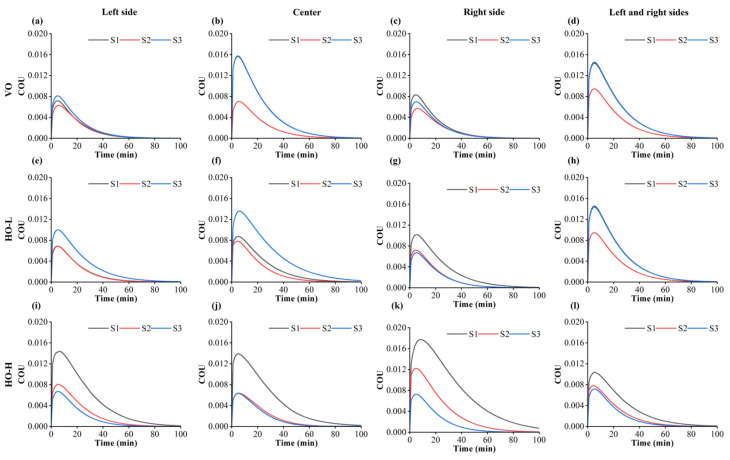
Variation in surface uniformity of thin layers of paddy at different outlet locations and drying time for different drying positions. (**a**–**d**) The airflow direction is VO and outlets are located on the left, center, and left and right sides, respectively. (**e**–**h**) The airflow direction is HO-L and different outlet locations. (**i**–**l**) The airflow direction is HO-H and different outlet locations.

As can be observed in Figure 14, the changes in COU occurred in two stages. First, in the initial stage, the edge material heated up quickly, and the edge heat was transferred inward for heat transfer. In addition, the diffusion and evaporation of moisture as the sample surface dried led to slower warming in the middle than at the edges, resulting in slower moisture removal [52]. This caused the COU to increase. After that, the paddy temperature reached thermal equilibrium with the air temperature, and the COU gradually decreased (Figure 14).

The location of the airflow outlet significantly influenced the temperature distribution of the paddy. In VO and HO-H, the results had smaller errors because the left and right outlets were two mirror image positions. However, the outlet position in HO-L had a greater effect on the uniformity of paddy drying. In HO-L, the highest peak value of the sample was 0.018. When the airflow direction was VO and the outlet direction was on the left and right sides, the average COU peak for the three samples was 0.007. The heat uniformity coefficient of the S2 paddy surface consistently remained lower than that of S1 and S3 (Figure 14a–d). Eddy currents caused localized areas of temperature variation, while airflow recirculation further disrupted uniform heating across the surface, contributing to temperature imbalances. After 50 min of drying, when the outlet was located at the left side or right side, the temperatures of the three groups of the paddy gradually approached thermal equilibrium with the air temperature. The drying time was 28.6% shorter than that of the other two groups.

### 4.5. Effect of Different Air Flow Directions on the Temperature Difference of Paddy

**Figure 15:**
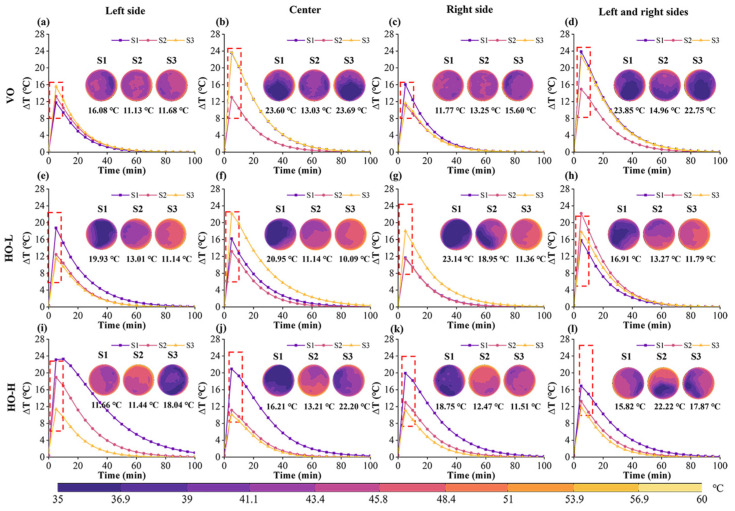
Distribution of temperature profiles and maximum temperature difference in paddy for different airflow directions and outlet positions. The red dashed box indicates ∆*T*. (**a**–**d**) The airflow direction is VO and outlets are located on the left, center, and left and right sides, respectively. (**e**–**h**) The airflow direction is HO-L and different outlet locations. (**i**–**l**) The airflow direction is HO-H and different outlet locations.

Figure 15 illustrates that the changes in ∆*T* on the surface of the paddy can be divided into two stages. The first stage involves an increase in ∆*T*, where the surface of the paddy begins to absorb heat, leading to a gradual rise in temperature. During the second stage, the surface temperature gradually approaches the air temperature, leading to a tendency for the internal and surface temperatures to equilibrate and causing a subsequent decrease in the temperature difference [53]. Notably, Δ*T* exhibited little variation when the airflow direction was VO and the outlet was at the left side or right side. The maximum temperature difference of the paddy reached 16.08 °C.

When the airflow direction was VO, the temperature distribution on the surface of S2 was more uniform than that of S1 and S3 (Figure 15a–d). Additionally, the outlet position significantly affected S1 and S3, with the maximum ∆*T* reaching 23.85 °C. When the outlets were positioned at the center and both sides, the ∆*T* of S1 was approximately 23.60 °C and 23.85 °C, respectively. The ∆*T* of S3 was approximately 23.69 °C and 22.75 °C, respectively (Figure 15b,d). The thick boundary layer needed to conduct heat inward, resulting in a slow rise in internal temperature.

When the airflow direction was HO-L, the ∆*T* between S3, S2, and S1 gradually increased (Figure 15e–h). The airflow rate on the windward side of S3 was large, and the temperature remained relatively stable, resulting in high heat transfer efficiency and improved uniformity with a smaller temperature difference. However, the airflow around S2 was still higher than that around S1, likely due to vortices and instabilities in the airflow through S3 and S2. The windward surface of S2 experienced more uniform heating in HO-H (Figure 15i–l). The surface temperatures of S1 and S3 were uneven due to surrounding airflow. The side near S2 had a higher temperature, while the side away from S2 had a lower temperature. As shown in Figure 15i, the formation of low-pressure regions and vortices on the S3 surface led to a significant reduction in heat transfer efficiency, creating a considerable temperature difference between the edge and the center of the surface.

## 5. Conclusions

This study developed a conjugate model for hot air drying of a three-dimensional paddy layer by coupling CFD with heat and mass transfer, accounting for the fluid domain around the paddy. The impact of various airflow directions, outlet locations, and different positions on the drying process was investigated. The feasibility of the simulation model was verified through a combination of experimental and simulation methods.

(1) The surface temperature distribution of the simulated paddy drying closely matched the experimental temperature curve. The *RMSE* of the simulated average temperature compared to the experimental values was relatively large, at 2.155, while the *RMSE* of the average moisture content among the three samples was smaller, at 0.252.

(2) Airflow direction, outlet location, and the obstruction caused by the paddy resulted in significant changes in velocity and in the temperature fields within the drying chamber. The airflow vortex structure caused the edge airflow velocity of the paddy to exceed that of the center. The temperature distribution was more uniform when the airflow direction was VO.

(3) The simulation results indicated that the edge effect on the temperature of the paddy in CD was pronounced. The surface temperature of the paddy layer was slightly weakened not only by the fluid but also by the evaporation of moisture. In comparison to HO-L and HO-H, when the outlet of the airflow direction VO was on the left or right side, the time required to reach thermal equilibrium between the paddy temperature and the air temperature was shortened by 28.6%, resulting in the best temperature uniformity among the three paddy samples.

The simulation model developed in this study can further optimize convective drying and combined convective drying, providing a reference for enhancing drying uniformity. Based on these results on drying uniformity, this research can provide an accurate approach for future studies on the changes in paddy quality after drying and the optimization of different drying process parameters.

## Figures and Tables

**Table 2:** Statistical parameters for simulation and experimentation of samples.

Samples	Temperature (°C)		Moisture Content (w.b.)	
	*RMSE*	*R* ^2^	*RMSE*	*R* ^2^
S1	2.285	0.964	0.241	0.995
S2	2.270	0.963	0.298	0.992
S3	2.279	0.963	0.256	0.994
S_ave_	2.155	0.967	0.252	0.995

## Data Availability

The original contributions presented in the study are included in the article, further inquiries can be directed to the corresponding author.

## Abbreviations

**Nomenclature**			
*c*	moisture concentration (mol/m^3^)	*R* ^2^	coefficient of determination
*c_0_*	initial moisture concentration (mol/m^3^)	*Re*	Reynolds number
*c_e_*	equilibrium moisture concentration (mol/m^3^)	R_eq_	distance or equivalent radius (m)
C_1_, C_2_	empirical constants	*RMSE*	root mean square error
*C_pair_*	specific heat capacity of air (J·kg^−1^K^−1^)	S1, S2, S3	Sample1, Sample2, Sample3
*C_pm_*	specific heat capacity of paddy (J·kg^−1^K^−1^)	*Sc*	Schmidt number
CD	convection drying	*Sh*	Sherwood number
CFD	computational fluid dynamics	*t*	time (min)
COU	coefficient of uniformity	*T*	temperature (°C)
*D*	diffusion coefficients (m^2^/s)	T_0_	initial temperature of paddy (°C)
D_0_	Arrhenius factor (m^2^/s)	*T_air_*	drying air temperature (°C)
*D_air_*	diffusivity of water in air (m^2^/s)	*T_max_*	maximum temperature (°C)
*D_eff_*	effective moisture diffusivity (m^2^/s)	*T_min_*	minimum temperature (°C)
E_a_	activation energy (J/mol)	*u*	air velocity vector (m/s)
*G_k_*	production of turbulent kinetic energy	VO	vertical orientation
*h_m_*	mass transfer coefficient (m/s)	∆*T*	difference in temperature (°C)
*h_t_*	heat transfer coefficient (W·m^−2^s^−1^)	Greek symbols	
HO-L	horizontal orientation with longitudinal arrangement of materials	*μ_air_*	viscosity of air (kg/m^3^)
HO-H	horizontal orientation with horizontal arrangement of materials	*μ_t_*	turbulent viscosity (kg/m^3^)
*H_vap_*	enthalpy of water evaporation (J/kg)	*κ*	kinetic energy
*k_g_*	thermal conductivity of paddy (W·m^−1^K^−1^)	*ε*	dissipation rate
*k_air_*	thermal conductivity of air (W·m^−1^K^−1^)	*σ*	Prandtl number
*MC*	moisture content (kg/kg, w.b.)	*ρ_air_*	density of air (kg/m^3^)
M_0_	initial moisture content (kg/kg, w.b.)	*ρ_m_*	density of paddy (kg/m^3^)
*M_e_*	equilibrium moisture content (d.b.)	Subscripts	
M_H2O_	molecular mass of water (kg/mol)	air	air
*M_wb_*	wet-basis moisture content (kg/kg, w.b.)	d.b.	dry basis
*n*	number of data	e	equilibrium
*Nu*	Nusselt number	exp	experiment
*Pr*	Prandtl number	sim	simulation
R	universal gas constant (J·mol^−1^K^−1^)	w.b.	wet basis
